# Tobramycin Clearance Is Best Described by Renal Function Estimates in Obese and Non-obese Individuals: Results of a Prospective Rich Sampling Pharmacokinetic Study

**DOI:** 10.1007/s11095-019-2651-2

**Published:** 2019-05-30

**Authors:** Cornelis Smit, Roeland E. Wasmann, Marinus J. Wiezer, Hendricus P. A. van Dongen, Johan W. Mouton, Roger J. M. Brüggemann, Catherijne A. J. Knibbe

**Affiliations:** 1 Department of Clinical Pharmacy, St. Antonius Hospital, Koekoekslaan 1, 3435 CM Nieuwegein, The Netherlands; 2 Department of Systems Biomedicine and Pharmacology, Leiden Academic Centre for Drug Research, Leiden University, Leiden, The Netherlands; 3 Department of Pharmacy, Radboud Institute for Health Sciences, Radboudumc, Nijmegen, The Netherlands; 4 Department of Surgery, St. Antonius Hospital, Nieuwegein, The Netherlands; 5 Department of Anesthesiology, St. Antonius Hospital, Nieuwegein, The Netherlands; 6 Department of Medical Microbiology and Infectious Diseases, Erasmus MC, Rotterdam, The Netherlands

**Keywords:** aminoglycosides, morbid obesity, pharmacokinetics, tobramycin

## Abstract

**Purpose:**

Tobramycin is an aminoglycoside antibiotic of which the 24 h exposure correlates with efficacy. Recently, we found that clearance of the aminoglycoside gentamicin correlates with total body weight (TBW). In this study, we investigate the full pharmacokinetic profile of tobramycin in obese and non-obese individuals with normal renal function.

**Methods:**

Morbidly obese individuals (*n* = 20) undergoing bariatric surgery and non-obese healthy volunteers (*n* = 8), with TBW ranging 57–194 kg, received an IV dose of tobramycin with plasma concentrations measured over 24 h (*n* = 10 per individual). Statistical analysis, modelling and simulations were performed using NONMEM.

**Results:**

In a two-compartment model, TBW was the best predictor for central volume of distribution (*p* < 0.001). For clearance, MDRD (de-indexed for body surface area) was identified as best covariate (*p* < 0.001), and was superior over TBW ((*p* < 0.05). Other renal function estimates (24 h urine GFR and de-indexed CKD-EPI) led to similar results as MDRD (all *p* < 0.001)).

**Conclusions:**

In obese and non-obese individuals with normal renal function, renal function estimates such as MDRD were identified as best predictors for tobramycin clearance, which may imply that other processes are involved in clearance of tobramycin *versus* gentamicin. To ensure similar exposure across body weights, we propose a MDRD-based dosing nomogram for obese patients.

**Electronic supplementary material:**

The online version of this article (10.1007/s11095-019-2651-2) contains supplementary material, which is available to authorized users.

## Introduction

The global prevalence of obesity and morbid obesity, which is commonly defined as a body mass index (BMI) over 30 and 40 kg/m^2^, respectively, is rapidly rising. In 2015, over 600 million adults were obese worldwide, accounting for 12% of the entire adult population (1). Due to physiological changes associated with obesity, such as an increase in fat and other tissue, differences in liver size, liver flow, liver enzyme activity and glomerular filtration rate (GFR), obesity-related changes in pharmacokinetic (PK) and/or pharmacodynamic (PD) parameters of drugs may be expected (2). However, the exact quantification of these changes in PK and PD is lacking for many drugs. This is of particular relevance for drugs for which a target concentration and/or exposure related to efficacy or safety has been identified, like in the case of aminoglycosides. These antibiotics, such as gentamicin and tobramycin, are used for the treatment of severe infections, with their efficacy being closely related to a (timely) attainment of an adequate plasma exposure (depicted by the 24-h area under the curve (AUC_24_) over the minimal inhibitory concentration (MIC) of the microbiological target (3–5). Since in the general population AUC_24_ closely correlates with the maximum plasma concentration (C_max_) and measurement of an AUC puts substantial burden to the treated patient, the C_max_ is often used as measure of efficacy with target values between 15 and 20 mg/L. Despite this approach that is used in clinical practice, the AUC_24_ is still considered the cornerstone PD-index for aminoglycoside effectivity and toxicity (5–7), with 75 mg*h/L being proposed as a pharmacodynamic target with an optimal effect and acceptable risk for toxicity (5). However, this is based on the assumption that MICs are not higher than 1 mg/L, whereas the wild-type population of most gram-negatives extend to 2 mg/L (5,8).

To date, in clinical practice tobramycin is dosed on a mg/kg basis. Clinicians may however be reluctant to use mg/kg dosing in (morbidly) obese patients, since high trough levels (i.e. >1 mg/L 24 h after dosing) are associated with side effects such as nephro- or ototoxicity (9,10). Therefore, over the past decades, several alternative body size descriptors to guide aminoglycoside dosing have been proposed, such as adjusted body weight (ABW) and lean body weight (LBW) (11–16). These dosing measures were mainly proposed to compensate for a body weight-related increase in volume of distribution (V_d_) which was found in these studies (11–16), with V_d_ being the parameter that determines C_max_. However, since not V_d_, but drug clearance drives the AUC, it is essential to clarify what body size descriptor or parameter best predicts clearance with increasing body weight. For the aminoglycoside gentamicin, we recently found that in obese individuals, TBW was the most predictive descriptor for clearance, albeit in a nonlinear manner (17). In the current prospective rich sampling study, we investigate the pharmacokinetics of tobramycin in morbidly obese and non-obese individuals with normal renal function (eGFR>60 ml/min), in order to investigate how tobramycin clearance and other PK parameters change in obesity. In line with our previous study on gentamicin PK in the obese, beside weight measures, other measures like renal function estimates were investigated as covariates. The results are used to guide dosing of tobramycin in (morbidly) obese individuals.

## Materials and Methods

This prospective observational study was registered in the Dutch Trial Registry (NTR6058), approved by the local human research and ethics committee and was conducted in accordance with the principles of the Declaration of Helsinki.

### Participants

Morbidly obese patients (BMI > 40 kg/m^2^ or > 35 kg/m^2^ with comorbidities) scheduled for bariatric surgery (laparoscopic gastric sleeve or gastric bypass) were considered for inclusion. In addition, a group of non-obese healthy volunteers (body mass index (BMI) 18–25 kg/m^2^) was included.

Participants were excluded when they had a known allergy to aminoglycosides, used potentially nephrotoxic medication in the week before surgery (such as lisdiuretics, vancomycin, ACE-inhibitors, non-steroid anti-inflammatory drugs), had a known renal insufficiency (eGFR <60 ml/min, using the Modification of Diet in Renal Disease (MDRD) (non-obese) or LBW in the Cockcroft Gault formula (obese) (18)), were pregnant or breastfeeding. Before inclusion, all participants provided written informed consent.

### Study Procedures

Twenty morbidly obese patients received 5 mg/kg LBW (calculated according to Janmahasatian (19)) tobramycin on the day of surgery as a single dose infused over 0.5 h, after which venous blood samples were collected at t = 5 min after end of infusion, followed by collections at t = 1, 1.5, 2, 2.5, 3.5, 4.5, 6, 12 and 24 h after start of infusion. 3 mL blood samples were collected in lithium-heparin tubes, centrifuged at 1900 g for 5 min, and plasma was stored at −80°C until analysis. Eight non-obese healthy volunteers received a single dose of 5 mg/kg TBW tobramycin, infused over 0.5 h, after which the same sampling scheme was employed.

In order to measure the glomerular filtration rate (GFR), urine was collected over 24-h on the study day and before and 24 h after administration of tobramycin, a blood sample was collected to measure serum creatinine. In addition, GFR was estimated (eGFR) as follows: (1) using the Cockcroft-Gault formula with LBW without correction for gender for obese and with TBW for non-obese individuals (CG-LBW) (18), (2) using the Modification of Diet in Renal Disease (MDRD) which was de-indexed for body surface area (BSA) by multiplying with individual BSA/1.73, and (3) Chronic Kidney Disease Epidemiology Collaboration (CKD-EPI) formula, also de-indexed for body surface area (BSA) by multiplying with individual BSA/1.73 (18). Equations for the different renal function estimates are shown in the supplemental material.

Total tobramycin plasma concentrations were measured using a commercially available, validated immunoassay kit (Cobas® TOBR2, Roche Diagnostics GmbH, Mannheim), with a lower limit of quantification (LLOQ) of 0.3 mg/L.

### Pharmacokinetic Analysis

For each individual, AUC_24_ was calculated using the trapezoidal rule. C_max_ was defined as the measured concentration 1 h after start of the 0.5-h infusion. Categorical data was analysed using Fischer Exact test, where continuous data is compared using the Wilcoxon Rank test.

Using all data, population pharmacokinetic modelling was performed with NONMEM 7.3 (ICON Development Solutions, Hanover, USA), Pearl-speaks-NONMEM (PsN) 4.6.0 and visualized using Pirana 2.9.7 (Pirana Software & Consulting BV), R 3.4.4 and GraphPad Prism 6.0 (GraphPad Software, La Jolla, USA) (20–22). Concentrations below LLOQ were retained in the dataset and analysed using the M3 method, where a likelihood for being below LLOQ was estimated for these concentrations (23). Discrimination between nested models was done by comparing the objective function value (OFV, −2 log likelihood) as obtained from the NONMEM output. A difference in OFV of 3.84, corresponding with a *p* value <0.05 for one degree of freedom, was considered statistically significant. In addition, goodness-of-fit plots (GOF, observed *versus* population and individual predicted values, individual weighted residuals *versus* time or population predicted values), prediction-variability corrected visual predictive checks (pvcVPC), precision of parameter estimates, shrinkage, and individual plots were examined for diagnostic purposes. One-, two- and three-compartment models were evaluated as structural models. Inter-individual variability (IIV) on the individual parameter estimate of the *i*th individual (θ_*i*_) was modelled according to Eq. (1):

1 $$ {\theta}_i={\theta}_{mean}\times {e}^{\eta_i} $$

where θ_mean_ is the population mean parameter value, η_*i*_ is a random variable for the *i*th individual with a mean of zero and variance of ω^2^, assuming log-normal distribution in the population. For residual variability a combined, proportional and additional error model was investigated, according to eq. (2):

2 $$ {Y}_{ij}={C}_{pred, ij}+\left({C}_{pred, ij}\times {\varepsilon}_1\right)+{\varepsilon}_2 $$

where Y_*ij*_ is the observed concentration, C_*pred,ij*_ the predicted concentration for the *j*th observation in the *i*th individual and ε_1_ and ε_2_ the proportional and additive errors, respectively, with a mean of zero and variance of σ^2^.

### Covariate Analysis

The influence of covariates was explored by plotting individual posthoc parameter estimates or the IIV estimates against individual covariate values. Covariates were TBW, LBW (calculated using the Janmahasatian formula) (19), ABW (calculated as ideal body weight (IBW) + 0.4 * (TBW-IBW) (11)), BMI, GFR, de-indexed MDRD, de-indexed CKD-EPI, CG-LBW, sex and age. Equations are summarized in the supplemental material. Continuous covariates were implemented using the following equations:

3 $$ {P}_i={P}_p\times {\left(\frac{ CO V}{{CO\mathrm{V}}_{standard}}\right)}^X $$

4 $$ {P}_i={P}_p\times \left(1+Z\times \left( COV-{COV}_{standard}\right)\ \right) $$

where *P*_i_ and *P*_p_ represent individual and population parameter estimates, COV represents the covariate, COV_standard_ represents a population standardized (e.g. 70 kg for TBW) or median value for the covariate, X represents the exponent for a power function and Z represents the relative change of the parameter in a linear covariate relationship. Linear covariate relationships were tested with a slope parameter Z using eq. (4) or by fixing the exponent X in eq. (3) to 1. In addition, the recently described function characterising the influence of TBW on gentamicin clearance (17), was evaluated for its performance for tobramycin (i.e. equation (3) using TBW as covariate with an exponent of 0.729), which is an approach that was applied before on aminoglycosides in neonates and children (24,25). Categorical covariates were entered into the model by calculating a separate pharmacokinetic parameter for each category of the covariate. After entering covariates separately into the model, their added value was statistically tested using the OFV. In addition, if applicable, it was evaluated whether the IIV for the parameter decreased upon inclusion of the covariate and whether the trend in the IIV *versus* covariate disappeared. In general, a forward inclusion (*p* < 0.05, OFV decrease >3.8) and backward deletion (*p* < 0.001, OFV decrease >10.8) strategy was employed for inclusion of covariate. Finally, earlier mentioned general diagnostics were taken into account.

### Internal Model Validation

pvcVPC’s were generated using PsN (*n* = 1000 datasets split for obese and non-obese) with prediction and variability correction. Bootstrap re-sampling (*n* = 1000, stratified on weight group, i.e. obese and non-obese) was performed to obtain confidence intervals for the parameters, as well as to assess the robustness of the model.

### Model-Based Simulations

Using the final PK model, Monte Carlo simulations were performed with interindividual and residual variability in 9.993 individuals with body weights uniformly distributed between 60 and 190 kg. Values for de-indexed MDRD were assigned to each individual using a normal distribution with separate mean and standard deviation (SD) for obese (mean: 137 ml/min, SD: 34) and non-obese (mean: 112 ml/min, SD: 23) groups, based on the distributions found in the ongoing AMIGO trial (Dutch Trial Registry NTR6058, *n* = 60 obese, *n* = 32 non-obese individuals,). Four dosing scenarios were simulated: (1) tobramycin 5 mg/kg TBW, (2) de-indexed MDRD based dosing using the relationship between clearance and MDRD as was found a-posteriori in the final PK-model, with 75 mg*h/L as target for the AUC_24_ (5) and (3) 5 mg/kg ABW. For comparison, (4) simulations using a dosing strategy based on the best function identified for TBW (Table III) were also performed. All infusions were simulated as single intravenous administrations given in 0.5 h.

## Results

### Demographics and Data

A total 20 obese and 8 non-obese participants were included in this study. Obese patients had a median TBW of 137.8 kg (range 103–194) *versus* 66.3 kg (range 57–91) in the non-obese group. Patient characteristics are shown in Table I. For each individual, 10 samples were obtained resulting in 280 tobramycin plasma concentrations in total. Of these, 23 (8.2%) were below LLOQ of 0.3 mg/L.

**Table I:** Summary of Patient Characteristics

	Morbidly obese (*n* = 20)	Non - obese (*n* = 8)	P value
Male/female	9/11	4/4	0.57
Age (years)	43.0 [27–54]	22.5 [20–25]	<0.001
Total body weight (TBW, kg)	137.8 [103–194]	66.3 [57–91]	<0.001
Lean body weight (LBW (19), kg)	69.3 [51–107]	49.7 [38–69]	0.0029
Body Mass Index (BMI, kg/m^2^)	41.9 [36–53]	22.2 [19–25]	<0.001
Glomerular filtration rate based on 24-h urine collection (GFR, ml/min)	163.3 [85–230]	124.7 [98–141]	0.031
Estimated GFR			
De-indexed Modification of Diet in Renal Disease (MDRD, ml/min)	127.5 [77–171]	102.6 [91–120]	0.031
De-indexed Chronic Kidney Disease Epidemiology Collaboration (CKD-EPI, ml/min)	138.0 [78–171]	109.4 [101–129]	0.050
Cockcroft Gault with LBW (19) (obese) or TBW (non-obese) (CG-LBW, ml/min)	116.4 [69–148]	119.8 [101–138]	0.40
Tobramycin dose (mg)	340 [240–480]	320 [280–440]	0.75

Data shown as median [range] unless otherwise specified

**Fig. 1:**
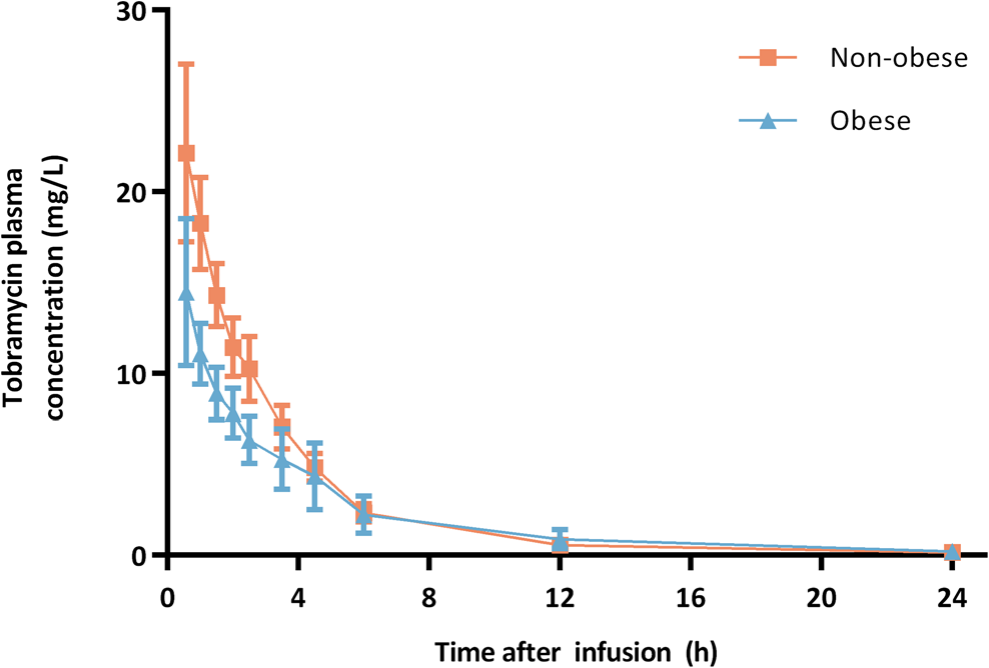
Mean ± SD tobramycin plasma concentrations *versus* time after end of infusion for obese (blue triangles, *n* = 20, dose: 5 mg/kg lean body weight) and non-obese individuals (orange squares, *n* = 8, dose: 5 mg/kg total body weight).

The mean measured tobramycin plasma concentrations for each timepoint are shown in Fig. 1. The AUC_24_ was significantly lower in the obese group receiving tobramycin as a single 5 mg/kg LBW dose compared to the non-obese control group receiving a 5 mg/kg TBW dose (mean 56.1 ± 16.3 mg*h/L *vs*. 70.0 ± 12.0 mg*h/L, *p* = 0.039). Also C_max_ levels were significantly lower in the obese individuals (mean 11.8 ± 2.8 mg/L *vs*. 18.3 ± 2.7 mg*h/L, *p* < 0.001). No nephrotoxicity (based on the RIFLE criteria (26)) was observed in any participant.

### Pharmacokinetic Analysis

A two-compartmental model with first-order elimination from the central compartment and a combined additional and proportional residual error model best described the data. IIV was implemented on clearance and central volume of distribution. Parameters of the structural model without covariates (base model) are shown in Table II.

**Table II:** Population Pharmacokinetic Parameters of the Base and Final Tobramycin Model and Results of the Bootstrap Analysis

	Base model (%RSE)		Final model (%RSE)		Bootstrap final model (*n* = 1000)		
						(95% Confidence interval)	
					Mean	Lower	Upper
V_c_ (L)	17.2	(7.3)	–				
V_c_ = Vc_70kg_ * (TBW/70)							
V_c 70 kg_ (L)	–		10.6	(5.9)	10.6	8.94	12.4
CL (L/h)	6.42	(4.3)	–				
CL = CL_MDRD 115_ * (1 + Z * (MDRD-115)							
CL_MDRD 115_ (L/min)	–		6.33	(2.4)	6.33	6.02	6.63
Z	–		0.00990	(3.9)	0.0100	0.0880	0.0122
V_p_ (L)	4.24	(15)	4.35	(5.6)	4.41	2.84	5.98
Q (L/min)	6.4	(5.1)	6.69	(1.6)	6.77	2.63	10.91
*Inter-individual variability (IIV, %)*							
V_c_	42.9	(9.3)	24.9^a^	(13)	24.1	14.9	30.8
CL	25.2	(14)	12.0^a^	(13)	11.7	7.90	14.5
*Residual variability*							
Proportional error	0.112	(12)	0.116	(11)	0.115	0.0880	0.141
Additive error (mg/L)	0.369	(13)	0.346	(11)	0.342	0.239	0.445
OFV	351.7		289.6		276.6	185.9	367.2

^a^η-shrinkage in the final model is 8% for IIV on CL and 6% for IIV on V_c_. *CL* Clearance from the central compartment, CL_MDRD 115_ Clearance from the central compartment for a person with a MDRD of 115 ml/min, *MDRD* De-indexed Modification of Diet in Renal Disease (in ml/min), *OFV* Objective Function Value, *Q* intercompartmental clearance between V_C_ and V_P,_ *RSE* Relative standard error, *TBW* Total body weight in kg, *V*_*c*_ Central volume of distribution, *V*_*c70 kg*_ Central volume of distribution for a 70 kg person

**Fig. 2:**
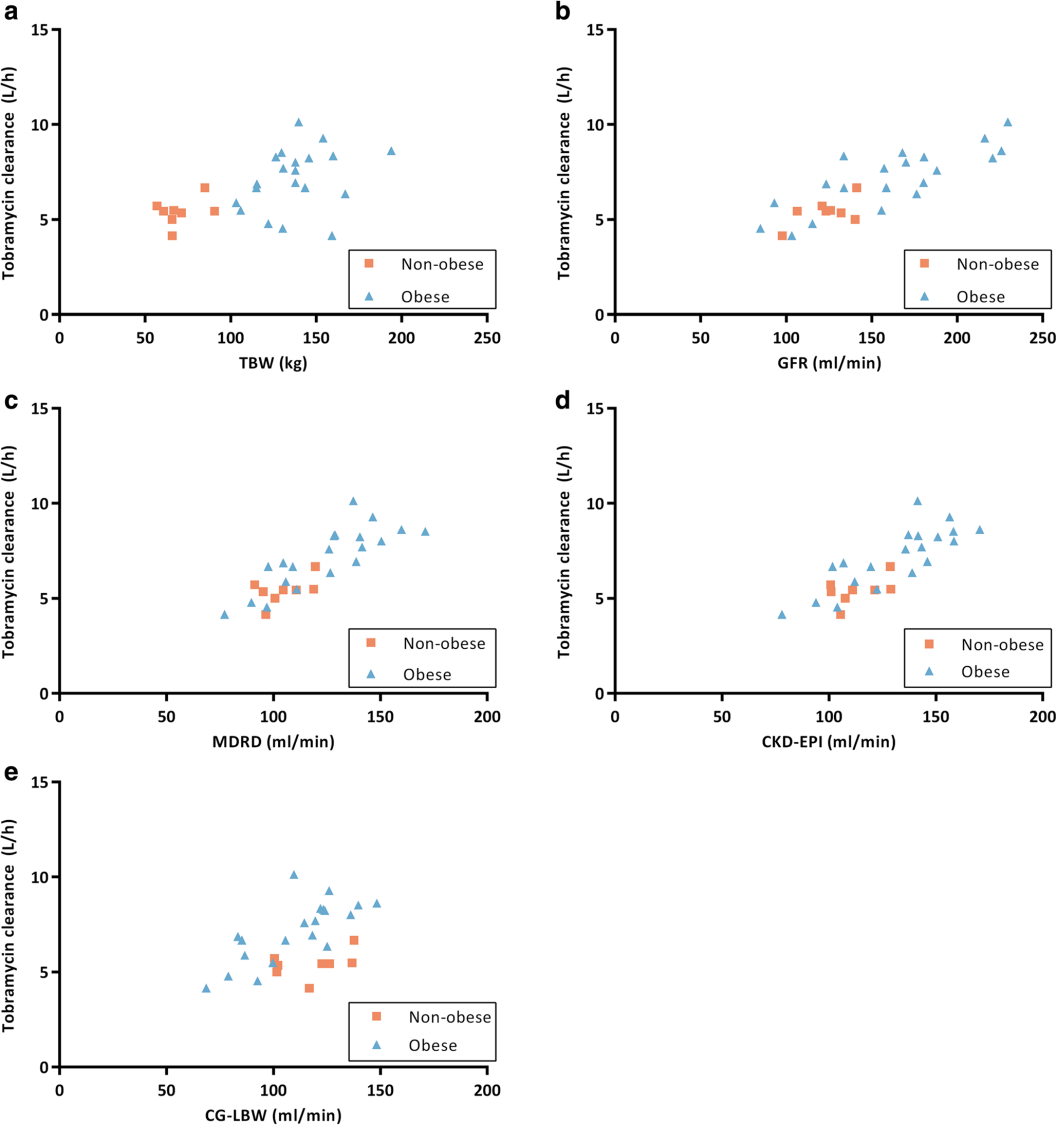
Individual posthoc clearance values for tobramycin (*n* = 28, in L/h) *versus* (**a**) total body weight (TBW), (**b**) 24-h urine glomerular filtration rate (GFR), (**c**) eGFR based on de-indexed Modification of Diet in Renal Disease (MDRD), (**d**) de-indexed Chronic Kidney Disease Epidemiology Collaboration (CKD-EPI) and (**e**) Cockcroft-Gault using LBW in obese and TBW in non-obese (CG-LBW). Obese individuals are shown in blue triangles, non-obese individuals in orange squares.

Exploration using scatter plots of individual posthoc parameter estimates and IIV against different covariates indicated TBW, ABW and LBW as candidate covariates for central volume of distribution, and de-indexed MDRD, de-indexed CKD-EPI, CG-LBW, GFR, TBW and LBW for clearance. Figure 2 shows the individual posthoc parameter estimate for clearance *versus* the different candidate covariates, showing particularly clear relationships for GFR, MDRD and CKD-EPI.

For central volume of distribution, TBW in a power function, LBW and ABW as linear covariates resulted in significant OFV drops (−25.9, −23.9 and − 29.2, respectively, all *p* < 0.001). As TBW gave the best GOF (populations predicted *versus* observed concentrations) with the least bias especially in higher concentrations (i.e. >12 mg/L), TBW was selected over ABW (*p* > 0.05). Inclusion of TBW on central volume of distribution resulted in a reduction of IIV from 42.9% to 25.1% (Table II).

The results of the covariate implementation on CL are shown in Table III. Table III shows that implementation of de-indexed MDRD, de-indexed CKD-EPI, and GFR resulted in the largest reduction in OFV, i.e. -36.3, −32.8 and − 32.3, respectively (all *p* < 0.001). GOF plots for all covariates were comparable, although all models seemed to slightly underpredict tobramycin concentrations below 10 mg/l in the non-obese individuals (data not shown). The addition of TBW to de-indexed MDRD as covariate for clearance improved this underprediction, however the limited reduction in OFV (i.e. -3.4 in OFV, *p* > 0.05) and only moderate improvement of GOF did not justify to include this extra parameter. Inclusion of de-indexed MDRD resulted in a reduction in IIV on clearance from 25.2% to 12.0% (Table II). Implementation of TBW instead of de-indexed MDRD, resulted in a power function on clearance with an estimated exponent of 0.42, and was inferior to implementation of de-indexed MDRD (i.e. −10.3 *versus* − 36.3 in OFV drop, *p* < 0.05, and a resulting drop in IIV on CL of 25.2% to 20.6% *versus* 12.0%, respectively). Implementation of the covariate relationship between TBW and clearance as found for gentamicin in similar study (17), i.e. a power relationship with an exponent of 0.729, resulted in an even smaller drop in OFV (i.e. -4.0, *p* < 0.05), with inferior GOF and only a very modest reduction in IIV from 25.2% to 23.4%. As final model, de-indexed MDRD was selected as covariate on clearance, since MDRD gave a significantly larger OFV reduction (*p* < 0.05) and better GOF compared to CKD-EPI, and since in clinical practice a serum creatinine based eGFR such as MDRD is more readily available than 24-h urine based GFR.

**Table III:** Impact of Different Covariates on Tobramycin Clearance (CL)

Model	Parameter relationship (subpopulation)	X (exponential) / Z (linear)	Number of parameters	OFV	ΔOFV^a^
TBW on V_c_	–	–	8	325.8	(reference)
TBW on CL	Exponential (all)	0.42	9	315.6	−10.3
TBW on CL^b^	Exponential (all)	0.729 FIX	9	321.8	−3.96
MDRD on CL	Linear (all)	0.0099	9	289.6	−36.2
CKD-EPI on CL	Linear (all)	0.0089	9	293.0	−32.8
GFR on CL	Linear (all)	0.0055	9	293.5	−32.3
CG-LBW on CL	Linear (obese)	0.0069	9	315.9	−9.88

^a^OFV drop relative to reference model (base model with TBW on V_c_)

^b^Covariate relationship for clearance and TBW as reported for gentamicin in similar study (17)

*CG-LBW* Cockcroft Gault using lean body weight for obese and total body weight for non-obese individuals, *CKD-EPI* De-indexed chronic Kidney Disease Epidemiology Collaboration, *CL* Clearance, *GFR* Glomerular Filtration Rate based on 24-h urine collection, *OFV* Objective Function Value, *MDRD* De-indexed modification of Diet in Renal Disease, *TBW* Total body weight, *V*_*c*_ central volume of distribution

**Fig. 3:**
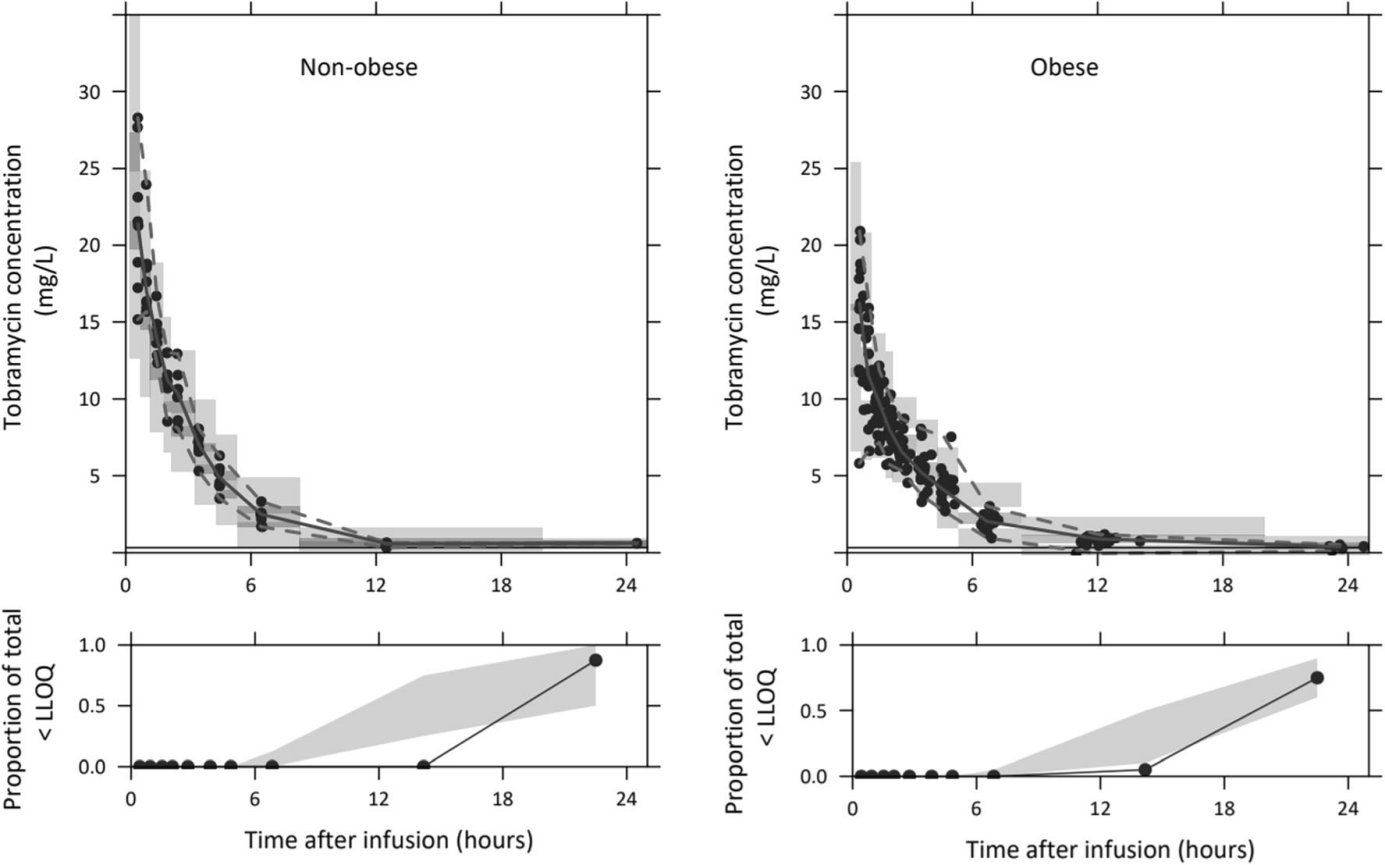
Prediction- and variability-corrected visual predictive checks (pvcVPC) of the final model for non-obese (upper left panel) and obese (upper right panel) individuals (*n* = 1000 simulations). The observed concentrations are shown as black circles, with median, 2.5th and 97.5th percentiles of the observed data as solid line, lower dashed line and upper dashed line, respectively. The grey shaded areas depict the 95% confidence intervals of the median (dark grey) and 2.5th and 97.5th percentiles (light grey) of predicted concentrations. Lower panels show the observed proportion below LLOQ (black dots), where shaded areas depict the 95% confidence interval of these proportion based on the simulated concentrations.

The GOF plots of the final covariate model are shown in Fig. S1 in the supplemental material and show that the model described the data well. The parameters of the final model with confidence intervals based on the bootstrap analysis are shown in Table II together with final equations for clearance and central volume of distribution. The results from the boostrap analysis (Table II) indicate a good precision and stability of the final model. The prediction-variability corrected visual predictive check (pvcVPC) shown in Fig. 3 indicates good validity of the final model, with median and 5th and 95th percentile of the observations being in concordance with the 95% confidence intervals of the simulations.

### Model-Based Simulations

**Fig. 4:**
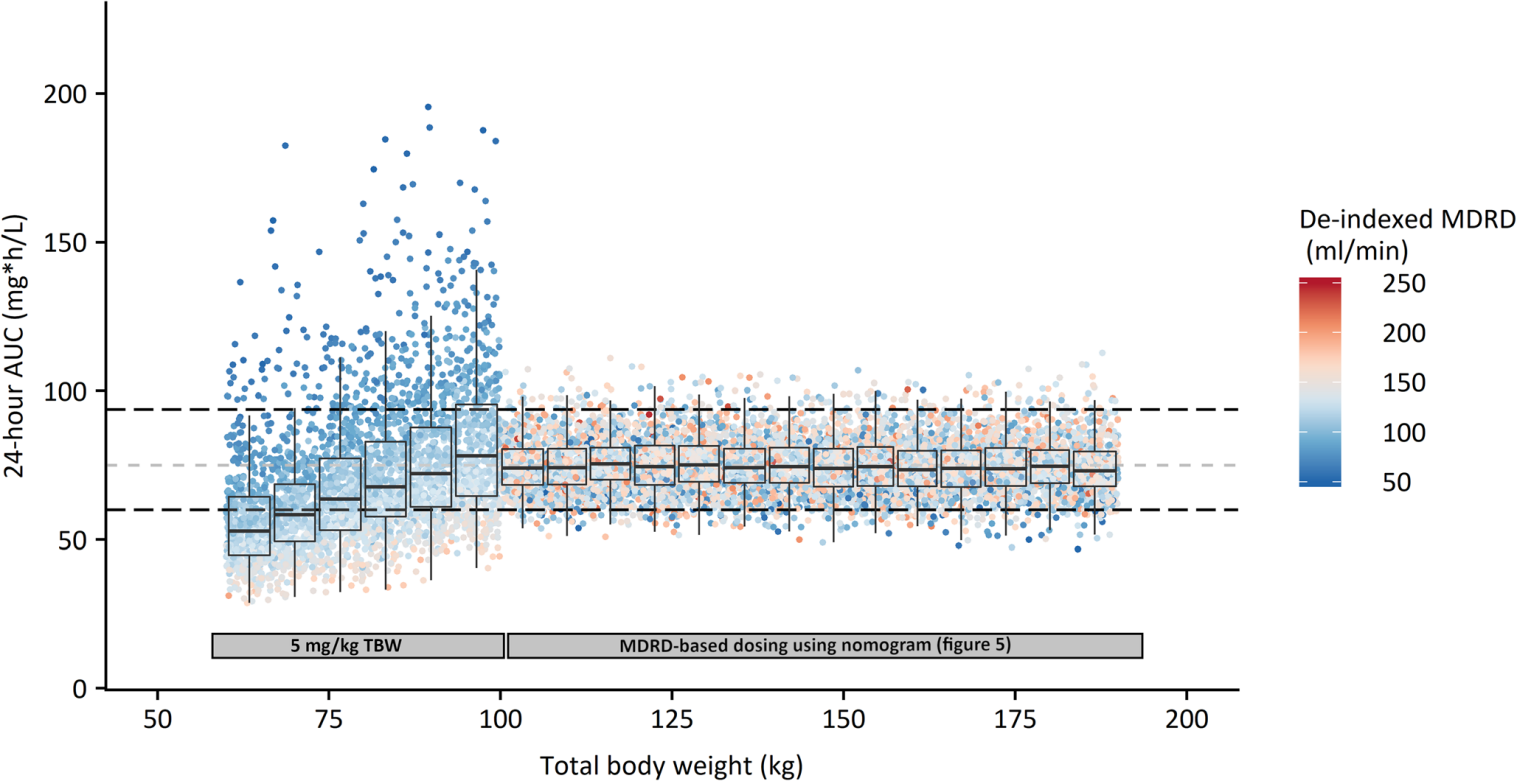
Monte Carlo simulations (*n* = 9.993) for individuals <100 kg receiving one IV dose of 5 mg/kg total body weight (TBW) tobramycin, and individuals >100 kg received a MDRD-based tobramycin dose using the nomogram in Fig. 5. Each dot represents the AUC_24_ (in mg*h/L) of one individual in the dataset, where the color shows the de-indexed MDRD in ml/min (calculated as MDRD * body surface area (BSA)/1.73) of this individual (ranging from dark blue to dark red with increasing MDRD). The boxplots represent median and interquartile range of AUC_24_ values within a specific total body weight subgroup. The grey dashed line shows the target AUC_24_ of 75 mg*h/L, black dashed lines show the 80–125% range (EMA acceptance criteria for bio-equivalence studies (27)) relative to this target value. *AUC* Area under the curve, *MDRD* Modification of Diet in Renal Disease *TBW* Total body weight.

**Fig. 5:**
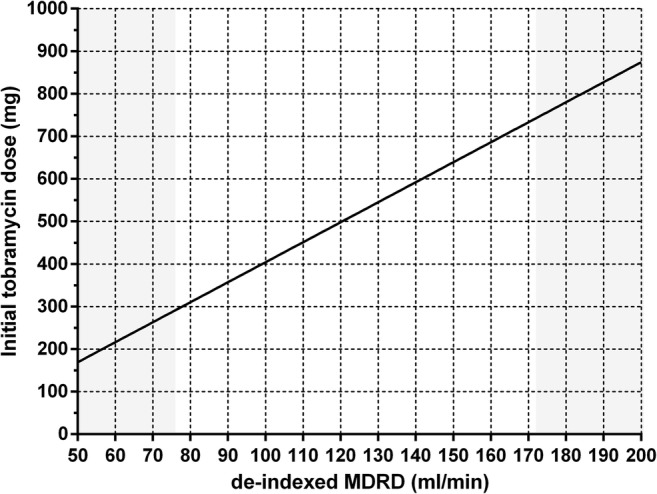
Dosing nomogram for tobramycin dose (in mg) based on the final tobramycin population PK model in non-obese and obese patients with body weights ranging from 57 to 194 kg and de-indexed MDRD values (calculated as MDRD * body surface area (BSA)/1.73) ranging from 77 to 171 ml/min, aiming for an AUC_24_ of 75 mg*h/L. The recommended tobramycin dose is calculated using equation: Dose (mg) = AUC_24,  target_ × 6.33 × (1 + 0.0099 × (MDRD − 115)). where AUC_24,target_ represents the target AUC_24_ in mg*h/L of 75 and MDRD represents the de-indexed MDRD in ml/min. Since the PK data consists of MDRD values from 77 to 171 ml/min, dose recommendations extrapolation to values outside these should be interpreted with caution (grey area in the nomogram). *MDRD* Modification of Diet in Renal Disease.

Figure 4 shows the individual (dots), median and interquartile range (boxplots) AUC_24_ values as obtained in the Monte Carlo simulations. Quantitative results are shown in Table S1 in the supplemental material. For individuals up to 100 kg (non-obese population), tobramycin was dosed as 5 mg/kg TBW. For obese individuals 100–190 kg, tobramycin was dosed using the nomogram depicted in Fig. 5, which is based on the relationship between clearance and MDRD as found in the final covariate model. The figure shows that when tobramycin is dosed as 5 mg/kg TBW, exposure increases with increasing body weight, with higher AUC_24_ values being observed in individuals with relatively low MDRD-values (<100 ml/min, dark blue dots). Median AUC_24_ per weight subgroup of non-obese individuals when receiving 5 mg/kg TBW increases from around 50 to 80 mg*h/L with increasing body weight. For individuals >100 kg, Fig. 4 shows that when a de-indexed MDRD-based dosing strategy is employed (using the nomogram in Fig. 5), no trend is visible with increasing body weight, with a median AUC_24_ tightly around 75 mg*h/L. In case the 5 mg/kg TBW dosing strategy was employed in obese individuals, an increase in both the mean and variability (range) of exposures is observed, with a median of around 150 mg*h/L for obese individuals weighing around 190 kg (Fig. S2B in supplemental material). When the MDRD-based dosing strategy is used in non-obese individuals as well, no remaining trend in this population is found (<100 kg, Fig. S2A in supplemental material). Finally, when dosing was performed based on scaled body weight (i.e. using 0.42 as exponent for TBW (Table III)) or ABW, no clear trends are visible in median exposure across body weights similar to MDRD-based dosing (Fig. S2C and D in supplemental material). However, in contrast to MDRD-based dosing, these do yield a substantial reduction in exposures within target in individuals with increased and decreased renal functions, respectively (Fig. S2C and D, Table S1).

## Discussion

In this report we studied the population pharmacokinetics tobramycin across body weights from 57 to 194 kg in individuals with a normal renal function. We show that with increasing body weight, tobramycin clearance is best predicted using a renal function estimate. In our data, this relationship between clearance and renal function was best described using de-indexed MDRD, although de-indexed CKD-EPI or GFR based on 24-h urine collection seem to lead to similar results. In order to reach the target exposure of 75 mg*h/L in individuals of varying weights, model-based simulations (Fig. 4) were performed showing that in obese individuals >100 kg tobramycin should be dosed using the proposed nomogram (shown in Fig. 5) based on the individuals de-indexed MDRD. Strong aspects of our study design are (1) the wide range of TBW in our study, including non-obese individuals and obese individuals up to 194 kg, (2) the rich sampling procedure up to 24 h post-infusion and, (3) the use of a modelling and simulation strategy that is nowadays seen as the gold standard by regulatory authorities for approval of new dose regimens (28).

The influence of obesity on aminoglycoside clearance has been reported in some studies over the years (11–13,15,16,29). Although in general these studies found an increase in clearance with increasing body weight, their results have to be interpreted with caution since individuals in most of these studies were only moderate obese compared to present-day standards with average body weights around 85–105 kg with standard deviations of ±12–18 kg (12,13,16). Moreover, analyses were often performed with sparse data collected up to only 8 h (11–13,15,16). These study designs limit the ability to properly assess drug clearance, particularly in view of the once every 24-h dosing that is currently in practice. Only few studies report on covariates that can be used to adequately predict aminoglycoside clearance in obese individuals. One clinical study by Pai *et al*. in 497 subjects (with 91 obese patients), report that both gentamicin and tobramycin clearance could be best predicted using unadjusted eGFR formula (either MDRD or CKD-EPI) rather than de-indexed eGFR functions or the CG formula (15). Our study found better predictions for eGFR over the CG-formula as well, although we found that de-indexed eGFR is preferred over the unadjusted estimates. A possible explanation for this difference might be that Pai *et al*. had to rely on sparse data, potentially making it more difficult to estimate individual tobramycin clearances. In addition, the authors used Mosteller’s equation for estimating BSA instead of the Dubois and Dubois formula as employed in our analysis, which may result in some differences. However, our results did not change significantly when the Mosteller’s equation was employed (data not shown). Lim *et al*. found in a retrospective study with 342 patients with ~30% being obese, that de-indexed eGFR outperformed their unadjusted counterparts in predicting aminoglycoside clearance (29). Leader *et al*. reported that ABW used in the CG equation is the best predictor for gentamicin clearance. Since this is an older study, no information is available on the performance of the eGFR formulas (12). Some other papers looked directly into predicting GFR in the obese population. These studies might be of relevance for our study, since in healthy adults, tobramycin clearance is shown to be primarily mediated through glomerular filtration (30). These papers generally agree that GFR can be best predicted using the de-indexed form of MDRD or CKD-EPI (31,32), or the CG formula with LBW or ABW (18,33,34). These conclusions are in line with our results, but should be translated to tobramycin clearance with caution since other (active) processes might be involved besides glomerular filtration when using GFR to predict clearance of a drug. In summary, it appears that most literature point to a renal function estimate to be most predictive for tobramycin clearance in obese individuals, although results from previous studies are conflicting as to how these renal function estimates should be corrected in obese individuals. The current study, with rich data collected in a wide range of body weights and (unimpaired) renal functions, in our opinion now definitively shows that de-indexed MDRD or CKD-EPI outperform body weight, the CG formula (using either TBW or LBW) and unadjusted renal function estimates in predicting tobramycin clearance in obese individuals.

Our results on tobramycin differ from results that we have found for gentamicin in a recently performed prospective pharmacokinetic study that studied a similar patient population in a similar study design (17). This study showed that the increase in gentamicin clearance was best described by TBW with an estimated allometric exponent of 0.73. In contrast to tobramycin, renal function estimates (eGFR or GFR based on 24-h urine collection) were inferior to TBW in predicting gentamicin clearance, despite the fact that in both studies individuals with a similar distribution in body weights and renal function (all >60 ml/min) were included. Interestingly, this finding has been reported before by other studies, describing stronger correlations between eGFR and drug clearance for tobramycin than gentamicin (15,29). To explain this difference between tobramycin and gentamicin, it could be hypothesized that transporters play a role. For gentamicin an increase in renal organic cation transporter 2 (OCT2) activity and consequently enhanced renal uptake has been reported that may contribute to increased gentamicin clearance in the obese. In an obese overfed mouse model, OCT2 activity increased with obesity, leading to increased renal accumulation of gentamicin (35). In addition, it is well established from studies with metformin, which is a well-known OCT2 substrate, that for OCT2 substrates drug clearance is influenced by altered OCT2 function. A human study showed that OCT2 genotypes associated with impaired activity led to a reduced apparent metformin clearance (CL/F) (36). Moreover, an increase in metformin CL/F was seen in obese adolescents compared to non-obese children, possibly due to an increase in renal OCT2-activity (37). In this light, the contrasting results on gentamicin and tobramycin clearance might be explained by a relatively higher dependence of gentamicin on OCT2-mediated renal uptake in favor of glomerular filtration. Although to our best knowledge, this never has been properly studied, this hypothesis is further substantiated by the observation that tobramycin accumulates less in the kidney compared to gentamicin and therefore might be less nephrotoxic (38). Further (preclinical) research seems warranted to clarify these differences between tobramycin and gentamicin PK based on the current study results.

An important question is what the target AUC_24_ is when treating patients with tobramycin. An AUC_24_ of 75 mg*h/L for pathogens with a MIC of 0,25–1 mg/L has been shown to be have the best balance between effectiveness and toxicity for aminoglycosides (5). Therefore, we provided a nomogram that can be used to determine the initial tobramycin dose for obese individuals based on the patient’s de-indexed MDRD targeting an AUC_24_ of 75 mg*h/L (Fig. 5). When this dose strategy is employed in the obese, a stable median AUC_24_ up to 190 kg without trends can be expected. In addition, outer ranges lie around ~75% to ~125% relative to the target of 75 mg*h/L (absolute 95% confidence interval of 57.4–93.5 and 56.9–92.8 mg * h/L for non-obese and obese individuals, respectively, visualized in Fig. 4). This is acceptable, considering the acceptance range of 80–125% as specified by the European Medicines Agency (EMA) for bio-equivalence studies (27). In contrast, when a 5 mg/kg TBW dose regimen is employed in obese individuals, the 95% confidence intervals lie between 22.2 mg*h/L and 184.1 mg*h/L, corresponding to 30% to 246% relative to the target AUC_24_. This high variability, which is most pronounced for the highest body weights of the obese population, can be explained by the fact that renal function is not taken into account in this strategy. Moreover, median AUC_24_ steadily increases with increasing body weight. In current daily practice, tobramycin is mostly dosed using ABW as is recommended by several papers, in order to maximize peak levels in obese individuals (11,39,40). However, like with TBW-based dosing, this approach does not consider variation in renal function. As such, our simulations show that this approach leads to a substantial reduction in the proportion of patients having an AUC within the target AUC_24_ compared to using the dose nomogram for the obese population (43.9% *versus* 93.6%). Therefore, even though inadequate target concentrations can be picked up by therapeutic drug monitoring that is usually performed after a one or more dosages, we do not recommend to use TBW or ABW-based dose regimens in obese individuals.

A few remarks should be made regarding the proposed nomogram. First, the dose nomogram shows dose recommendations for de-indexed MDRD values ranging from 30 to 250 ml/min. However, our PK-model is based on a dataset with MDRD values of 77 to 171 ml/min. Dose recommendations outside of this MDRD-range should therefore be interpreted with caution in clinical practice. Second, the AUC-target of 75 mg*h/L used in the nomogram is based on an AUC/MIC ratio of 75, with a corresponding MIC ≤1 mg/L, as has been proposed earlier (5). However, it is known that the wild-type population of most gram-negatives extends to 2 mg/L (8). Therefore, higher dosages might be necessary to cover the whole range of pathogens with MIC values up to 2 mg/L. Third, our study was specifically designed to obtain dose recommendations for obese individuals. A mg/kg-based dosing is already a widely accepted strategy for non-obese individuals. The proposed nomogram is expected to lead to an adequate exposure in the non-obese population as well (as shown in Fig. S3B in the supplemental material). Despite this, our simulations of a 5 mg/kg TBW dose (Fig. 4) show that in non-obese individuals, this strategy generally results in considerable variability. Last, after determining the initial tobramycin dose, we recommend that subsequent dosages should always be individualized by therapeutic drug monitoring, preferably with a limited sampling strategy in combination with model informed precision dosing based on Bayesian PK-software that is capable of translating the measured tobramycin concentrations to an individualized dose prediction (41).

Several limitations may apply to our study. First, we only included relatively healthy obese and non-obese individuals with an estimated renal function >60 ml/min/1.73 m^2^. Therefore, extrapolation of our study results to critically-ill patients with or without renal impairment should be done with caution, since critical illness can have an additional impact on PK. Secondly, obese study participants underwent bariatric surgery during the PK study, which might influence the PK results. However, since these surgeries in our hospital are very short (<1 h), and performed laparoscopically with minimal blood loss (<50 mL), we expect this impact to be negligible.

## Conclusion

In conclusion, we found that in non-obese and obese patients up to 194 kg, tobramycin clearance shows an important relation with renal function estimates. In obese individuals, de-indexed MDRD was superior over TBW in predicting tobramycin clearance. In order to yield similar exposure across body weights, we therefore propose that the tobramycin dose in individuals >100 kg should be based on de-indexed MDRD. To aid the clinician in finding the optimal dose, we provide a dose nomogram that can be used to determine the correct initial tobramycin dose by integrating MDRD and target AUC.

## Electronic supplementary material

ESM 1 (DOCX 521 kb)

## Abbreviations

(pvc)VPC: (prediction-variability corrected) Visual Predictive Check
ABW: Adjusted Body Weight
AUC: Area Under the Curve
AUC_24_: 24 h Area Under the Curve
BMI: Body Mass Index
BSA: Body Surface Area
CG: Cockcroft Gault
CKD-EPI: Chronic Kidney Disease Epidemiology Collaboration
GFR: Glomerular Filtration Rate
GOF: Goodness-of-fit
IBW: Ideal Body Weight
IIV: Inter-individual variability
LBW: Lean Body Weight
MDRD: Modification of Diet in Renal Disease
MIC: Minimal Inhibitory Concentration
OFV: Objective Function Value
TBW: Total Body Weight
