# Supplementary material for: Tobramycin Clearance Is Best Described by Renal Function Estimates in Obese and Non-obese Individuals: Results of a Prospective Rich Sampling Pharmacokinetic Study
Source: Pharm Res. 2019 May 30;36(8):112. doi: 10.1007/s11095-019-2651-2 (PMC6542779; doi:10.1007/s11095-019-2651-2)
Supplement: Supplementary file 1 — (DOCX 521 kb) [file 11095_2019_2651_MOESM1_ESM.pdf]

Title

Tobramycin clearance is best described by renal function estimates in obese and non-obese individuals: results of a prospective rich sampling pharmacokinetic study

Authors

Cornelis Smit, Roeland E. Wasmann, Marinus J. Wiezer, Hendricus P.A. van Dongen, Johan W. Mouton, Roger J.M. Brüggemann, Catherijne A.J. Knibbe.

Supplemental data

Equations for different covariates used in this study

Body weight descriptors

TBW (total body weight) =	Total body weight (kg)
BMI (body mass index, kg/m^2) =	TBW (kg)/((Length (m)^2)
BSA (body surface area, m^2) =	TBW (kg)$^{0.425}$ x (Length (cm)$^{0.725}$ x 0.007184
IBW (kg) =	50 (or 45.5 if female) + 2.3 x (Length (cm) x 0.3937-60)
ABW (kg) =	IBW (kg) + (0.4 x TBW-IBW)
	If TBW<IBW, TBW is used as ABW
LBW (kg) =	9270 x TBW / (6680+216 * BMI) if male
	9270 x TBW / (8780+244 * BMI) if female

Renal function estimates

MDRD (ml/min/1.73m^2) =	186.3 x (creatinine (mcmol/l)/88.4)$^{-1.154}$ x AGE (years) $^{-0.203}$ x 0.742 (if female) x 1.210 (if black)
De-indexed MDRD (ml/min) =	MDRD * BSA/1.73
CKD-EPI (ml/min/1.73 m^2) =	141 x min(creatinine (mg/dl)/k,1)^a x max(Scr/k,1)$^{-1.209}$ x 0.993^{age} x 1.018 (if female) x 1.159 (if black)
	k = 0.7 (females) or 0.9 (males)
	a = -0.329 (females) or -0.411 (males)
	min = minimum of creatinine/k or 1
	max = maximum of creatinine/k or 1
De-indexed CKD-EPI (ml/min) =	CKD-EPI * BSA/1.73
CG-LBW (ml/min) =	(140 – age (years)) x LBW (kg) / (F x creatinine (mcmol/l)
	F = 0.85 (females) or 0.81 (males)
CG-TBW (ml/min) =	(140 – age (years)) x total body weight (kg) / (F x creatinine (mcmol/l)
	F = 0.85 (females) or 0.81 (males)
GFR (ml/min) =	1000 x creatinine$_{urine}$ (mmol/l) / creatinine$_{serum}$ (mcmol/l) x volume$_{urine}$ (ml) / collection time (hours)

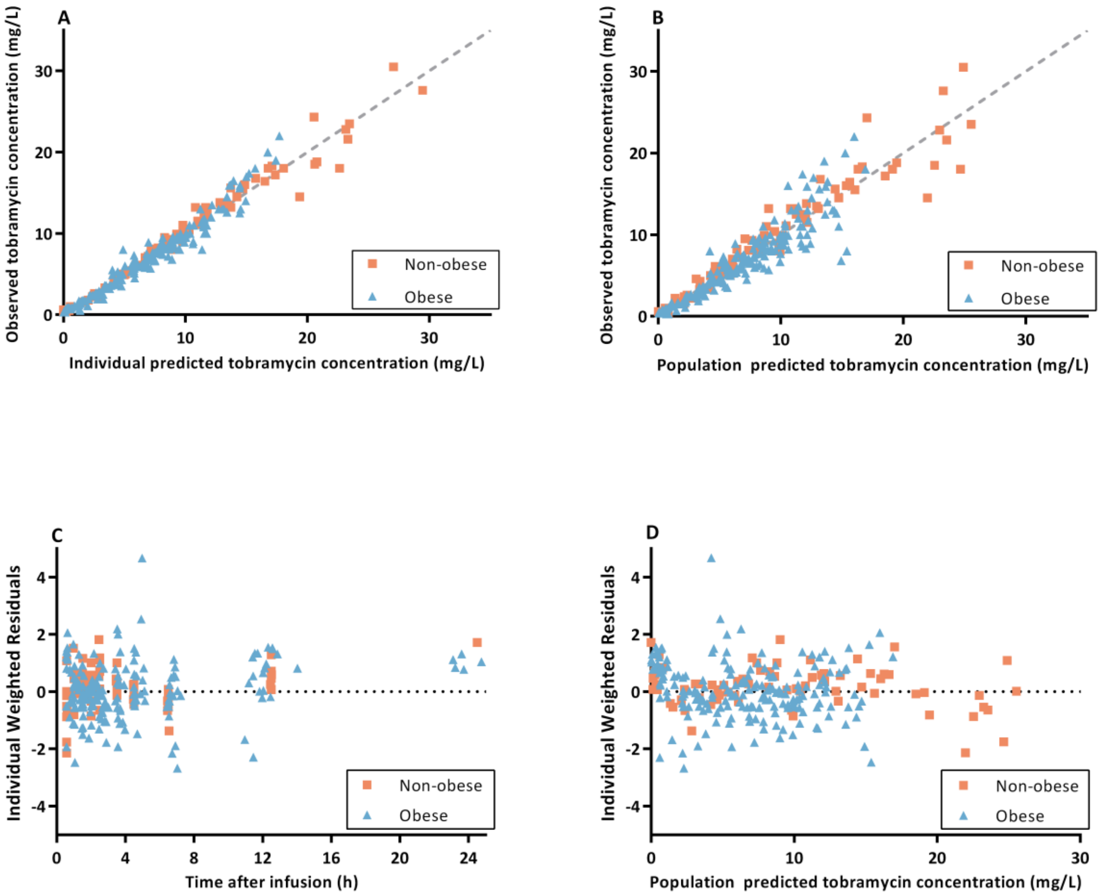

Figure S1. Goodness-of-fit plots of the final model for morbidly obese individuals (n = 20, blue triangles) and non-obese individuals (n = 8, orange squares): A) observed versus individual predicted tobramycin concentrations, B) observed versus population predicted tobramycin concentrations, C) individual weighted residuals versus time after start of infusion and D) versus population predicted tobramycin concentrations. The dashed lines in plots (A) and (B) represent the line of identity (x = y).

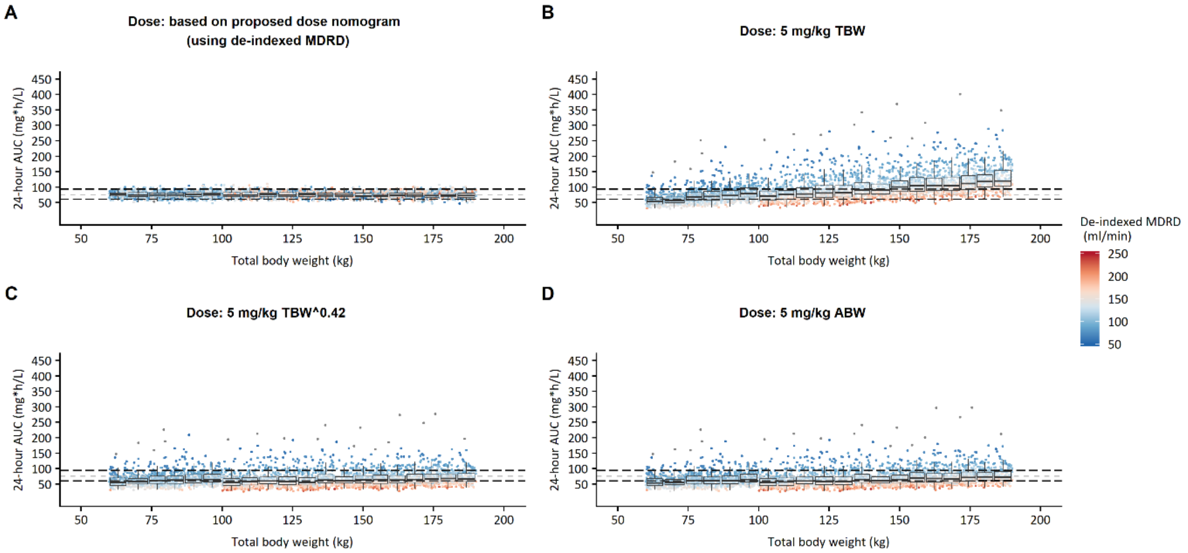

Figure S2. Monte Carlo simulations (n = 9.993) for individuals receiving (A) a MDRD-based tobramycin dose using the nomogram in Figure 5, (B) 5 mg/kg total body weight (TBW), (C) 5 mg/kg TBW scaled with exponent 0.42 (dose weight = 70 * (TBW/70)^0.42) or (D) 5 mg/kg adjusted body weight (ABW = Ideal Body Weight (IBW) + 0.4 * (TBW-IBW)) tobramycin. Each dot represents the AUC_{24} (in mg*h/L) of one individual in the dataset, where the color shows the de-indexed MDRD in ml/min (calculated as MDRD * body surface area (BSA)/1.73) of this individual (ranging from dark blue to dark red with increasing MDRD). The boxplot represents median and interquartile range of AUC_{24} values within a specific total body weight subgroup. The grey dashed line shows the target AUC_{24} of 75 mg*h/L, black dashed lines show the 80%-125% range (EMA acceptance criteria for bio-equivalence studies (1)) relative to this target value. *ABW* Adjusted body weight *AUC* Area under the curve, *MDRD* Modification of Diet in Renal Disease *TBW* Total body weight.

(1) Committee for Medicinal Products for Human use (CHMP) - European Medicines Agency (EMA). Guideline on the investigation of bioequivalence - CPMP/EWP/QWP/1401/98 Rev. 1. 2010.

Table S1 Results of Monte Carlo Simulations (n=9.993) with different dose regimens

**Simulated dose**	**Within target[b] (%)**		**AUC_{24h} (mg * h/L)**					
			**Median**		**95% CI lower limit**		**95% CI upper limit**	
	**Non-obese[c]**	**Obese[c]**	**Non-obese[c]**	**Obese[c]**	**Non-obese[c]**	**Obese[c]**	**Non-obese[c]**	**Obese[c]**
Nomogram (MDRD-based)	93.4	93.6	75.0	74.2	57.4	56.9	93.5	92.8
5 mg/kg TBW	48.0	40.4	65.0	94.6	23.3	22.2	116.8	184.1
5 mg/kg $TBW^{0.42}$ [a]	44.6	41.8	60.9	61.6	24.1	18.8	105.7	115.5
5 mg/kg ABW	40.2	43.9	58.8	63.5	23.3	18.4	102.3	120.3

[a] Dose weight = 70 * $(TBW/70)^{0.42}$

[b] Target is defined as 80%-125% relative to 75 mg*h/L (EMA acceptance criteria for bio-equivalence studies (1)).

[c] Non-obese individuals: subgroup with total body weight 60 – 100 kg, Obese individuals: subgroup with total body weight 100 – 190 kg.

ABW Adjusted body weight *AUC* Area under the curve, *MDRD* Modification of Diet in Renal Disease *TBW* Total body weight.

(1) Committee for Medicinal Products for Human use (CHMP) - European Medicines Agency (EMA). Guideline on the investigation of bioequivalence - CPMP/EWP/QWP/1401/98 Rev. 1. 2010.

95% CI 95% confidence interval *ABW* Adjusted body weight AUC_{24} 24 hour Area under the curve, *MDRD* Modification of Diet in Renal Disease *TBW* Total body weight.
